# The transcription factor FOXO4 is down-regulated and inhibits tumor proliferation and metastasis in gastric cancer

**DOI:** 10.1186/1471-2407-14-378

**Published:** 2014-05-28

**Authors:** Linna Su, Xiangqiang Liu, Na Chai, Lifen Lv, Rui Wang, Xiaosa Li, Yongzhan Nie, Yongquan Shi, Daiming Fan

**Affiliations:** 1 State Key Laboratory of Cancer Biology & Xijing Hospital of Digestive Diseases, The Fourth Military Medical University, 127 Changle Western Road, Xi’an, Shaanxi Province 710032, People’s Republic of China; 2 Department of Radiology, Xijing Hospital, Fourth Military Medical University, 127 Changle Western Road, Xi’an, Shaanxi Province 710032, People’s Republic of China

**Keywords:** FOXO4, Gastric cancer, Proliferation, Metastasis, EMT

## Abstract

**Background:**

FOXO4, a member of the FOXO family of transcription factors, is currently the focus of intense study. Its role and function in gastric cancer have not been fully elucidated. The present study was aimed to investigate the expression profile of FOXO4 in gastric cancer and the effect of FOXO4 on cancer cell growth and metastasis.

**Methods:**

Immunohistochemistry, Western blotting and qRT-PCR were performed to detect the FOXO4 expression in gastric cancer cells and tissues. Cell biological assays, subcutaneous tumorigenicity and tail vein metastatic assay in combination with lentivirus construction were performed to detect the impact of FOXO4 to gastric cancer in proliferation and metastasis in vitro and in vivo. Confocal and qRT-PCR were performed to explore the mechanisms.

**Results:**

We found that the expression of FOXO4 was decreased significantly in most gastric cancer tissues and in various human gastric cancer cell lines. Up-regulating FOXO4 inhibited the growth and metastasis of gastric cancer cell lines in vitro and led to dramatic attenuation of tumor growth, and liver and lung metastasis in vivo, whereas down-regulating FOXO4 with specific siRNAs promoted the growth and metastasis of gastric cancer cell lines. Furthermore, we found that up-regulating FOXO4 could induce significant G1 arrest and S phase reduction and down-regulation of the expression of vimentin.

**Conclusion:**

Our data suggest that loss of FOXO4 expression contributes to gastric cancer growth and metastasis, and it may serve as a potential therapeutic target for gastric cancer.

## Background

Although the incidence of gastric cancer(GC) is declining, it remains the fourth most common cancer and second leading cause of cancer-related death worldwide [1]. The key molecules involved in cell proliferation and metastasis in GC progression may aid in clinical diagnosing or predicting the progression of this disease.

Tumor growth and metastasis depend on various factors, including transcription factors [2-5]. The FOXO transcription factors family comprises four highly related members: FOXO1, FOXO3, FOXO4, and FOXO6 [6-8]. In recent years, FOXO have been shown to play crucial roles in a plethora of cellular processes, including proliferation, apoptosis, differentiation, stress resistance, and metabolic responses [9], and may therefore be promising targets for new medications in the field of oncology [10,11].

Our previous results demonstrated that the FOXO4 mRNA expression level was dramatically down-regulated in lymph node-positive colorectal carcinoma tissues compared to lymph node-negative tissues, suggested it may function as a negative regulator of the metastasis of colorectal carcinoma [12]. However, the expression and function of FOXO4 in gastric cancer were not known yet. The aim of our work has been to investigate the possible role of FOXO4 in gastric cancer carcinogenesis. Here, we report that FOXO4 repress cell proliferation and metastasis in gastric cancer by the regulation G1 cell-cycle arrest and vimentin.

## Methods

### Tissue specimens

For tissue specimens, all patients provided informed consent to use excess pathological specimens for research purposes. The protocols used in this study were approved by the hospital’s Protection of Human Subjects Committee. The use of human tissues was approved by the institutional review board of the Fourth Military Medical University and conformed to the Helsinki Declaration, as well as local legislation. Patients providing samples for the study signed informed consent forms.

### Immunohistochemistry

Immunohistochemical staining was performed using the the avidin-biotin complex immunoperoxidase method. The primary antibody against FOXO4 (1:100, ab63254, Abcam) diluted in PBS containing 1% (wt/vol) bovine serum albumin (BSA). Negative controls were performed by replacing the primary antibody with pre-immune mouse serum. Images were obtained under a light microscope (Olympus BX51, Olympus, Japan) equipped with a DP70 digital camera. The observer was blinded to the identity of the samples when scoring immunoreactivity.

### Evaluation of staining

For evaluation of the cell staining, the sections were examined by two independent pathologists without prior knowledge of the clinic-pathological status of the specimens. Cells that were stained brown were considered to be positive. The expression of FOXO4 was evaluated according to the ratio of positive cells per specimen (R) and staining intensity (I). The ratio of positive cells per specimen was scored as follows: 0 for staining of < 1%, 1 for staining of 2% to 25%, 2 for staining of 26% to 50%, 3 for staining of 51% to 75%, and 4 for staining of > 75% of the cells examined. The intensity was graded as follows: 0, no signal; 1, weak staining; 2, moderate staining; and 3, strong staining. A total score (R × I) of 0 to 12 was finally calculated and graded as negative (−score: 0–2) or positive (+, 3–12).

### Tissue collection

Tissue arrays were purchased from the Aomei company(Aomei C0124H,AM01C09,Aomei Biotechnology Co. Ltd., Xi’an, China) (Additional file 1: Table S1 and Additional file 2: Table S2). For the western blot analysis, GC tissues and adjacent nontumorous tissues were obtained from eight patients who had undergone surgery at the Department of General Surgery in our hospital. All cases of GC and normal gastric mucosa were clinically and pathologically proven. The protocols used in the studies were approved by the Hospital’s Protection of Human Subjects Committee. Patients who contributed fresh surgical tissue for the study had signed informed consent forms.

### RNA extraction and real-time PCR

Total RNA from the cells was extracted using Trizol (Invitrogen, Carlsbad, CA), and cDNA was synthesized using the Prime Script RT reagent kit (TaKaRa Biotechnology, Dalian, China) according to the manufacturer’s recommendations. A Light Cycler Fast Start DNA Master SYBR Green I System (Roche, Basel, Switzerland) was used for the real-time PCR. GAPDH mRNA was used as the internal control, and the reaction mix without the template DNA was used as the negative control. All of the samples were measured independently three times. The primer sequences were as follows: GAPDH: (forward) 5′-TGGTGAAGACGCCAGTGGA-3′ and (reverse)5′-GCACCGTCAAGGCTGAGAAC-3′; FOXO4: (forward) 5′-CTTTCTGAAGACTGGCAGGAATGTG-3′ and (reverse) 5′-GATCTAGGTCTATGATCGCGGCAG-3′; E-cadherin: (forward) 5′-GAGTGCCAACTGGACCATTCAGTA-3′and (reverse) 5′- AGTCACCCACCTCTAAGGCCATC-3′; and Vimentin: (forward) 5′-CAGGCAAAGCAGGAGTCCAC -3′and (reverse) 5′-GCAGCTTCAACGGCAAAGTTC -3′. All real-time PCR reactions were performed in triplicate.

### Oligonucleotide construction and lentivirus production

Three pairs of siRNA oligonucleotides targeting FOXO4 were synthesized by GenePharma Co., Ltd. The GAPDH sequences were used as a positive control. An unrelated sequence was used as a negative control (provided by GenePharma). The sequences were as follows: FOXO4 siRNA oligo-1: 5′-CGCGAUCAUAGACCUAGAUTTAUCUAGGUCUAUGAUCGCGTT-3′ (sense); FOXO4 siRNA oligo-2: 5′-CAGCUUCAGUCAGCAGUUATTUAACUGCUGACUGAAGCUGTT-3′ (sense); FOXO4 siRNA oligo-3: 5′-GUGACAUGGAUAACAUCAUTTAUGAUGUUAUCCAUGUCACTT-3′ (sense); GAPDH siRNA oligo (positive control): 5′-GUAUGACAACAGCCUCAAGTT-3′ (sense); and negative control: 5′-UUCUCCGAACGUGUCACGUTT-3′ (sense).

According to the manufacturers’ instructions, FOXO4 siRNA oligos were transfected into cells using the siRNA-Mate™ reagent (GenePharma Ltd., Shanghai, China). After cultured for 2 to 3 days, total RNA and protein were extracted. For stable transfection, a lentiviral overexpression vector (Lenti-FOXO4) was constructed (Shanghai GeneChem Co., Ltd., Shanghai, China). Using a GV166-puro Vector (GeneChem Co., Ltd., Shanghai, China), a lentiviral vector that expressed GFP alone (LV-control) was used as a negative control (NC).

### Western blot

Equal amounts of proteins were separated using sodium dodecyl sulfate–polyacrylamide gel(SDS-PAGE) electrophoresis and transferred to a nitrocellulose membrane (Bio-Rad, Hercules, CA). FOXO4 rabbit polyclonal antibody (Abcam, 1:500), CyclinD1 rabbit polyclonal antibody (ImmunoWay, 1:1000), β-actin mouse monoclonal antibody (Sigma,1:2,000), E-cadherin and Vimentin rabbit polyclonal antibody (Santa Cruz, CA, 1:1000) antibodies were used for the western blot experiments.

### Cell proliferation assay

The 3-[4,5-dimethylthiazol-2-yl]-2,5-diphenyl-tetrazolium bromide (MTT) assay was performed to evaluate the speed of cell proliferation,and was performed according to standard procedures. Each cell line was detected in triplicate.

### Migration and invasion assay

Transwell migration assays were performed in modified Boyden chambers (Transwell; Corning Inc. Lowell, MA, USA) at a density of 5 × 10^3^ cells per well. After 24 h of incubation at 37°C, the cells on the lower surface of the wells were fixed with 4% paraformaldehyde, stained with 1% crystal violet, and counted.

### High-content screening assay

Cell motility was surveyed using a Cellomics Array Scan VTI 1700 plus (Thermo Scientific, USA). In brief, cells in the log phase were harvested and plated into 96-well plates (5 × 10^3^ cells/well). After overnight culture at 37°C for adhesion, the culture medium was replaced with serum-free RPMI1640 medium, and the culture was continued for an additional 24 h. Then, cells were washed twice with ice-cold PBS and stained with Hoechst 33342 for 15 min in an incubator. Subsequently, the cells were again washed twice with ice-cold PBS and exposed to different treatments. Cell motility was detected using the Cellomics Array Scan VTI 1700 plus (Thermo Scientific) according to the manufacturer’s protocol (each group included five repeated wells).

### Confocal microscopy

For confocal microscopy experiments, cells were grown on Lab-Tek 24-well chamber slides (Thermo Fisher Scientific, USA). After overnight culturing, the cells were fixed, washed, and permeabilized with 0.3% Triton X- 100 in PBS for 10 min. Then,the cells were incubated with primary antibodies against E-cadherin and vimentin (dilution 1:300, Abcam) overnight at 4°C. The cells were also incubated with Cy3-conjugated anti-rabbit IgG (dilution 1:200 (Jackson Immuno Research, West Grove, PA, USA) for 1 h at room temperature in the dark. The cell nucleus was counterstained using DAPI for 5 min. Fluorescence was monitored and photographed with a confocal microscope (Thermo Fisher Scientific, USA).

### Animal studies

For animal research, nude mice 4 to 6 weeks of age were purchased from the Animal Center of the Chinese Academy of Science (Shanghai, China) and maintained in laminar flow cabinets under specific pathogen-free conditions. All procedures for animal experimentation were performed in accordance with the Institutional Animal Care and Use Committee guidelines of the Experiment Animal Center of the Fourth Military Medical University.

### Tumorigenicity in nude mice

Logarithmically growing cells were harvested using trypsin and washed twice with PBS. Then, 2 × 10^6^ cells in 0.2 ml were injected subcutaneously into the right upper back region of the mice. Four weeks after inoculation, tumor-bearing mice were sacrificed, and the size of the tumor was determined by caliper measurement of the subcutaneous tumor mass. Each experimental group contained 6 mice. Two independent experiments were performed, and they yielded similar results.

### Tail vein metastatic assay

Approximately 2 × 10^6^ cells were suspended in 0.2 ml of sterile PBS and injected into the tail veins of 10 mice. The mice were then monitored for tumor volume and overall health, and their lungs and livers were regularly observed using imaging microscopy.

### Statistical analysis

All statistical analyses were performed using SPSS 17.0 statistical software (SPSS, Inc., Chicago, Illinois). Variables with a P value less than 0.05 were considered to be statistically significant. *χ*^2^ tests were used to evaluate the significance of differences in FOXO4 expression frequency between GC tissues and adjacent nontumorous gastric tissues. The *t*-test (a one-way ANOVA test) was performed to evaluate the significance of the difference between cell proliferation, plate clones, and migration assays. Overall survival curves were plotted using the Kaplan-Meier method and were evaluated for statistical significance using a log-rank test(the Mann–Whitney *U* test and Kruskal-Wallis H test were adopted for other data).

## Results

### Expression of FOXO4 is down-regulated in GC tissues and cell lines

**Figure 1:**
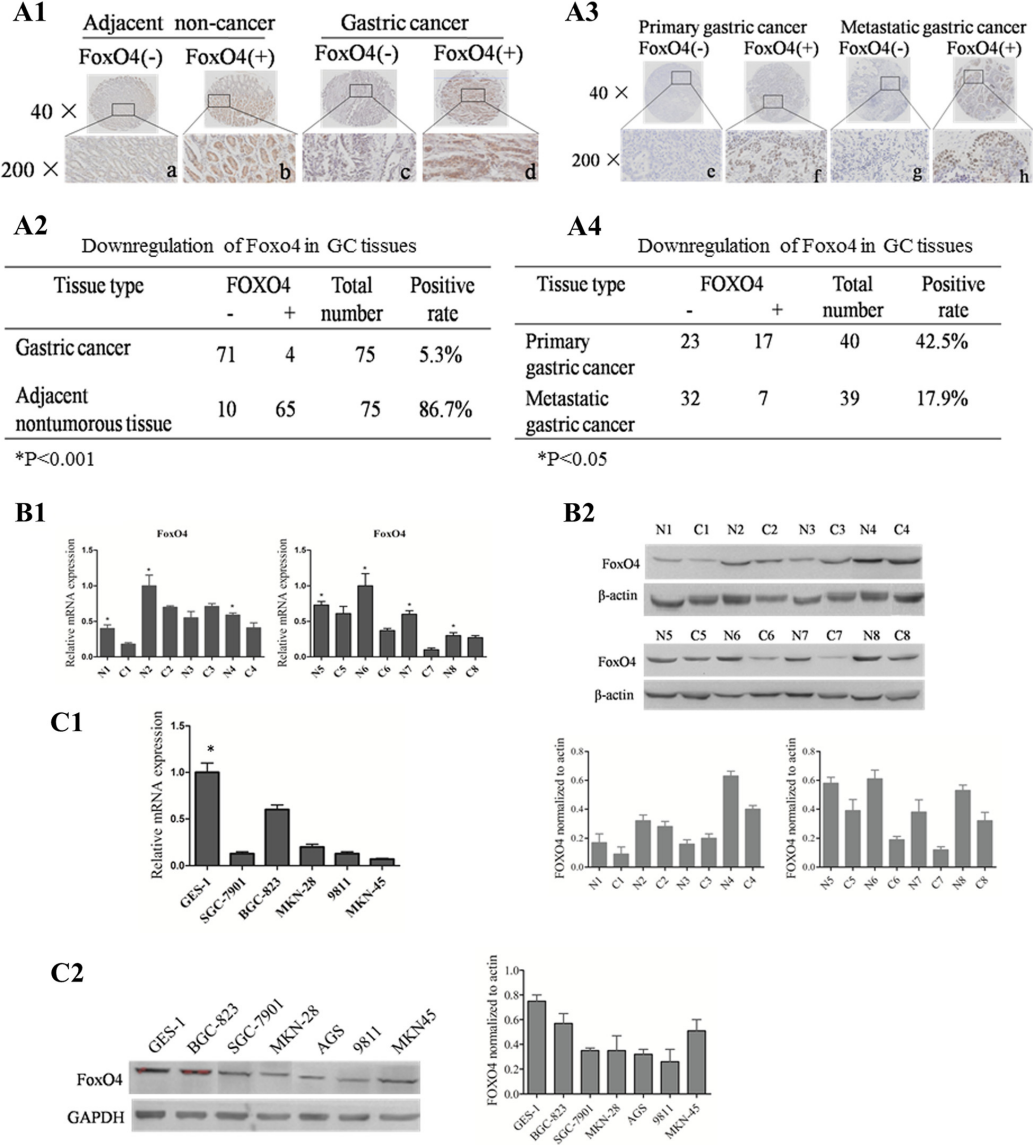
**FoxO4 is significantly down-regulated in GC tissues and cell lines. (A1)** IHC analysis of FOXO4 expression in 75 paired GC and adjacent non-tumorous tissues. **(A2)** Statistical analysis of FOXO4 expression in GC tissues and adjacent non-tumorous stomach tissues. **(A3)** Representative FOXO4expression in primary and metastatic GC tissues detected by IHC methods. **(A4)** Statistical analysis of FOXO4 expression between GC tissues with and without node metastasis. **(B1-B2)** Real-time PCR and western blot analysis of FOXO4 expression in 8 pairs of GC and adjacent non-tumorous tissues. **(C1-C2)** Real-time PCR and western blotting analysis of FOXO4 expression in different GC cell lines.

To examine whether the FOXO4 expression was altered in GC, the expression and subcellular localization of FOXO4 were studied in a tissue microarray of 75 paired GC samples by using an immunohistochemical assay. FOXO4 was mainly expressed in the nuclei of epithelial cells located in the gastric glands of nontumorous tissues (Figure 1A1), but a small amount was localized to the cytoplasm. The FOXO4 staining in epithelial cells from GC samples was weak. However, the FOXO4 staining in nontumorous tissues (NT) was consistently stronger than that of the GC samples, and there was a significant difference between the staining results of the GC and NT samples (Figure 1A2) (P < 0.05).We next measured the FOXO4 level in an independent tissue microarray panel containing 40 primary GCs and corresponding lymph node metastasis specimens. Overall, GCs showed a lower expression level of FOXO4 in metastatic lesions compared to the corresponding primary tumor samples (Figures 1A3-A4).The expression levels of FOXO4 were also examined by western blot and RT-PCR in GC and adjacent normal tissues obtained from eight patients (Figure 1B). In seven of the eight cases, FOXO4 was found to have reduced expression in cancerous tissues, consistent with the results from the immunohistochemistry analysis.We further compared the relative FOXO4 mRNA and protein expression levels among 6 different GC cell lines (BGC-823, SGC7901, MKN28, AGS, 9811, and MKN45) and the immortal gastric epithelial cell line GES-1. Again, FOXO4 was expressed at a relatively lower level in all 6 GC cell lines compared to the normal immortal gastric mucosal epithelial GES-1 cell line (Figure 1C). These results suggest that FOXO4 may play a suppressive role in gastric carcinogenesis.

### FOXO4 inhibits GC proliferation in vitro and induces cell cycle arrest in the G0/G1 phase

**Figure 2:**
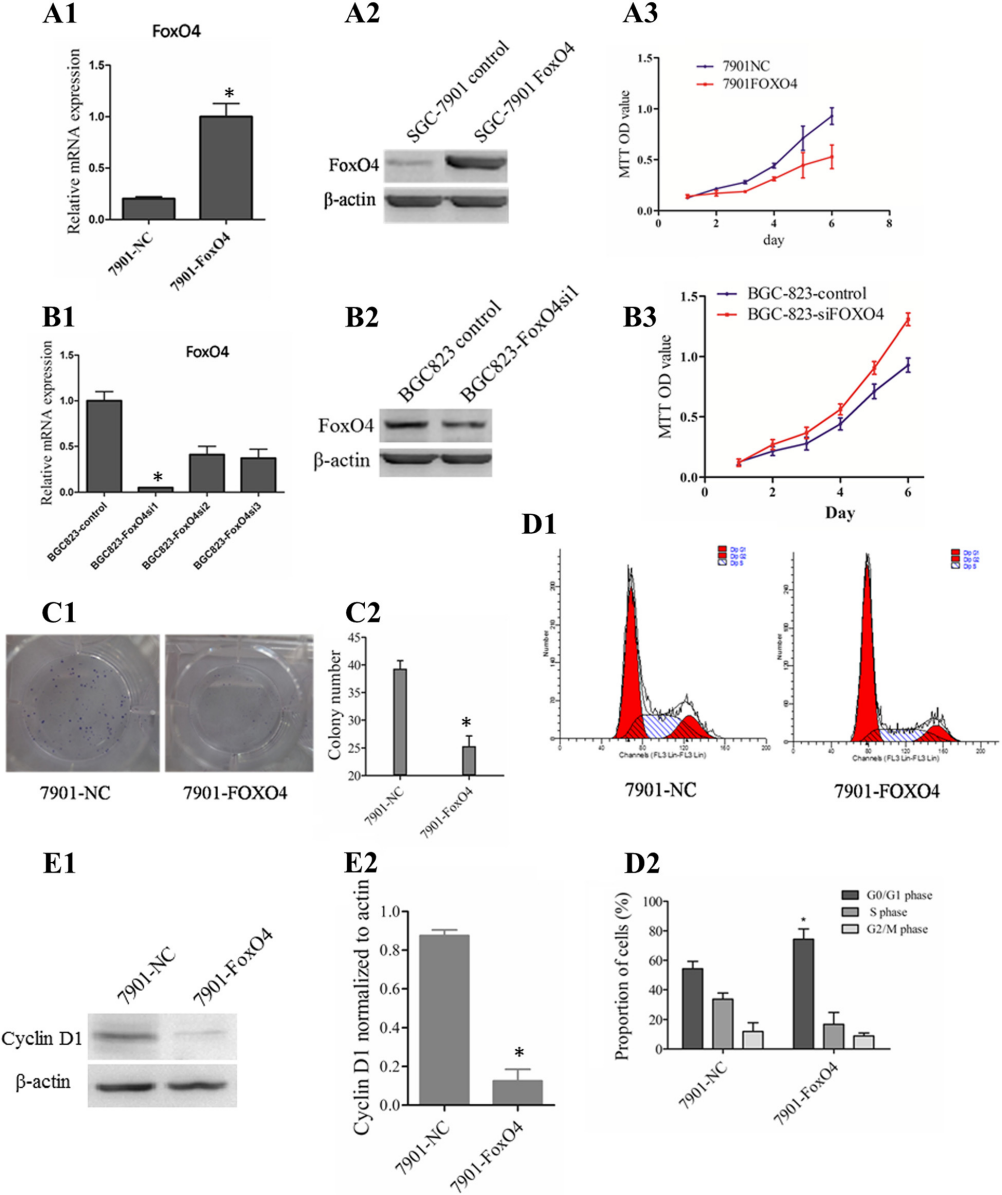
**Effect of FOXO4 on regulating GC cell proliferation. (A1-A2)** Relative expression of FOXO4 in SGC-7901 cells transfected with LV-FOXO4 or LV-control, which was confirmed by real-time PCR and western blot analysis. The values represent the means from three separate experiments, and the error bars represent the SEM (**P < 0.01). **(A3)** The proliferation rates of cells were measured using the MTT assay. The values represent the means from three separate experiments, and the error bars represent the SEM (*P < 0.05). **(B1-B2)** Relative expression of FOXO4 in BGC-823 cells transfected with FOXO4 oligo nucleotide inhibitor or oligo nucleotide control, which was confirmed by real-time PCR and western blot analysis. The values represent the means from three separate experiments, and the error bars represent the SEM (**P < 0.01). **(B3)** The proliferation rates of cells were measured using the MTT assay. The values represent the means from three separate experiments and the error bars represent the SEM (*P < 0.05). **(C1-C2)** Colony formation of SGC07901 cells transfected with LV-FOXO4 and LV-control was carried out by seeding cells onto plates for 2 weeks, and the number of colonies was then counted. The values represent the means from three separate experiments, and the error bars represent the SEM (*P < 0.05). **(D1-D2)** Cell cycle distribution of SGC-7901 cells transfected with LV-FOXO4 or LV-control. Cell cycle analysis was performed 24 h after transfection. The cell cycle distribution was calculated and expressed as the mean ± SD of three separate experiments. *P < 0.05. **(E1-E2)** Relative expression of CyclinD1 in SGC-7901 cells transfected with LV-FOXO4 or LV-control, which was confirmed western blot analysis. The values represent the means from three separate experiments, and the error bars represent the SEM(*P < 0.05).

To investigate the role of FOXO4 in GC growth, we established two stable cell lines (denoted SGC7901-FoxO4 and SGC7901-NC) after infection with the LV- FoxO4 or LV-NC lentivirus, respectively. After repeated puromycin selection, RT-PCR and a western blot analysis confirmed that SGC7901-FoxO4 showed higher FOXO4 expression compared to SGC7901-NC (Figure 2A1-A2). The MTT assay showed that up-regulation of FOXO4 expression significantly inhibited the proliferation of GC cells (Figure 2A3, P < 0.01).In contrast, the BGC823 cell line, which has relatively higher endogenous expression, was transiently transfected with FOXO4 siRNA or the negative control. Three pairs of siRNA oligonucleotides targeting FOXO4 were synthesized and transfected into BGC823 cells (BGC823-FOXO4si1, BGC823-FOXO4si2, and BGC823-FOXO4si3), and cells transfected with siRNA oligo negative control were labeled BGC823-siNC. qRT-PCR and western blot showed that siRNA oligo number 1 was the most effective, so, this construct was selected for further study (Figure 2B1-B2). Accordingly, the growth curves indicated that down-regulating the expression of FOXO4 resulted in increased proliferation among GC cells (Figure 2B3).We also performed a plate colony formation assay. These results revealed that SGC7901-FOXO4 cells produced fewer cell colonies compared to SGC7901-NC control cells (Figure 2C1-C2, P < 0.05). Next, we used FACS analysis to examine the effects of FOXO4 on the cell cycle. SGC7901-FOXO4 cells displayed significant G1 arrest and S phase reduction (Figure 2D1-D2), which indicated that FOXO4 inhibited GC proliferation as the result of G1 cell-cycle arrest. To reinforce this observation, we detected the expression of CyclinD1 which is a marker of G1 phase with western blot, it showed that CyclinD1had a relatively higher expression in the 7801-NC cell line than the 7901-FOXO4 cells (Figure 2E1-E2).

### FOXO4 inhibits the migration and invasion of GC cells in vitro

**Figure 3:**
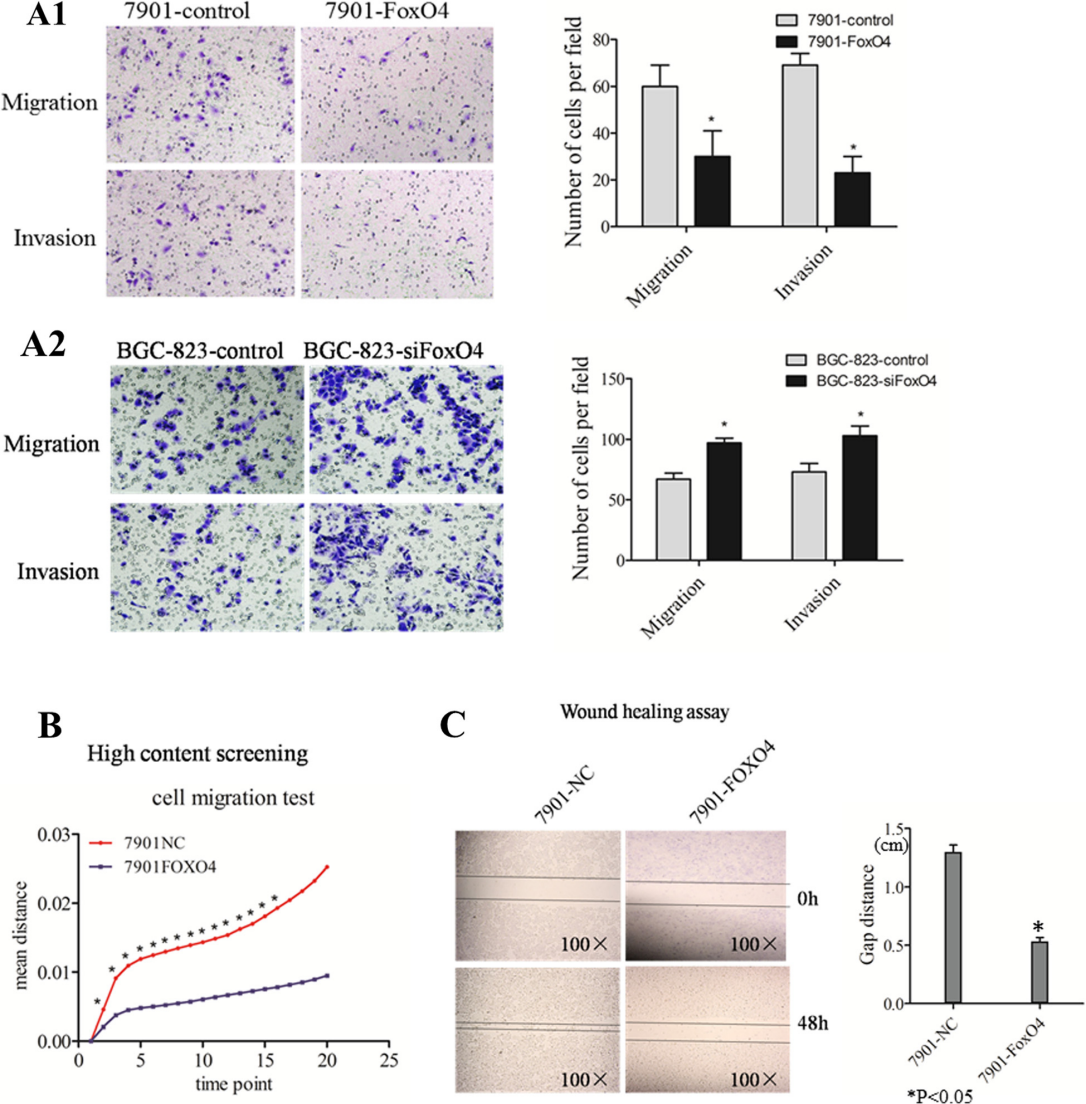
**Effect of FOXO4 in regulating GC cell metastasis. (A1-A2)** Up-regulation of FOXO4 expression in LV-FOXO4 cells decreased SGC-7901 cell migration and invasion in vitro, whereas the inhibition of FOXO4 expression using the oligo nucleotide inhibitor of FOXO4 enhanced BGC-823 cell migration and invasion. **(B)** Cell migration capacity was evaluated by performing a high-content assay in SGC-7901 cells transfected with LV-FOXO4 or LV-control. *P < 0.05 **(C)** Cell migration capacity was also tested by performing a wound-healing assay in SGC-7901 cells transfected with LV-FOXO4 or LV-control. *P < 0.05.

To evaluate the influence of FOXO4 on GC migration and invasion, we next evaluated the effect of FOXO4 expression on the invasive and migratory abilities of GC cells using in vitro transwell assays. The results showed that the migration and invasion of SGC-7901-FOXO4 cells were both notably reduced in comparison to SGC-7901-NC control cells (Figure 3A1). In contrast, depletion of FOXO4 significantly promoted cell migration and invasion in BGC823 cells compared to control cells (Figure 3A2). Furthermore, the high-content screening assay showed the motility speed of SGC-7901-FOXO4 cells is significantly lower than SGC-7901-NC cells,15/19 time points clearly showed the motility speed of SGC-7901-FOXO4 cells is lower than SGC-7901-NC cells (Figure 3B). Additionally, wound-healing assays showed that SGC-7901-FOXO4 cells closed wounds more slowly than SGC-7901-NC cells (Figure 3C) (P < 0.05). Together, these results indicated that FOXO4 significantly impaired GC cell migration and invasion in vitro.

### FOXO4 up-regulation inhibits tumorigenesis and metastasis of GC cells in vivo

**Figure 4:**
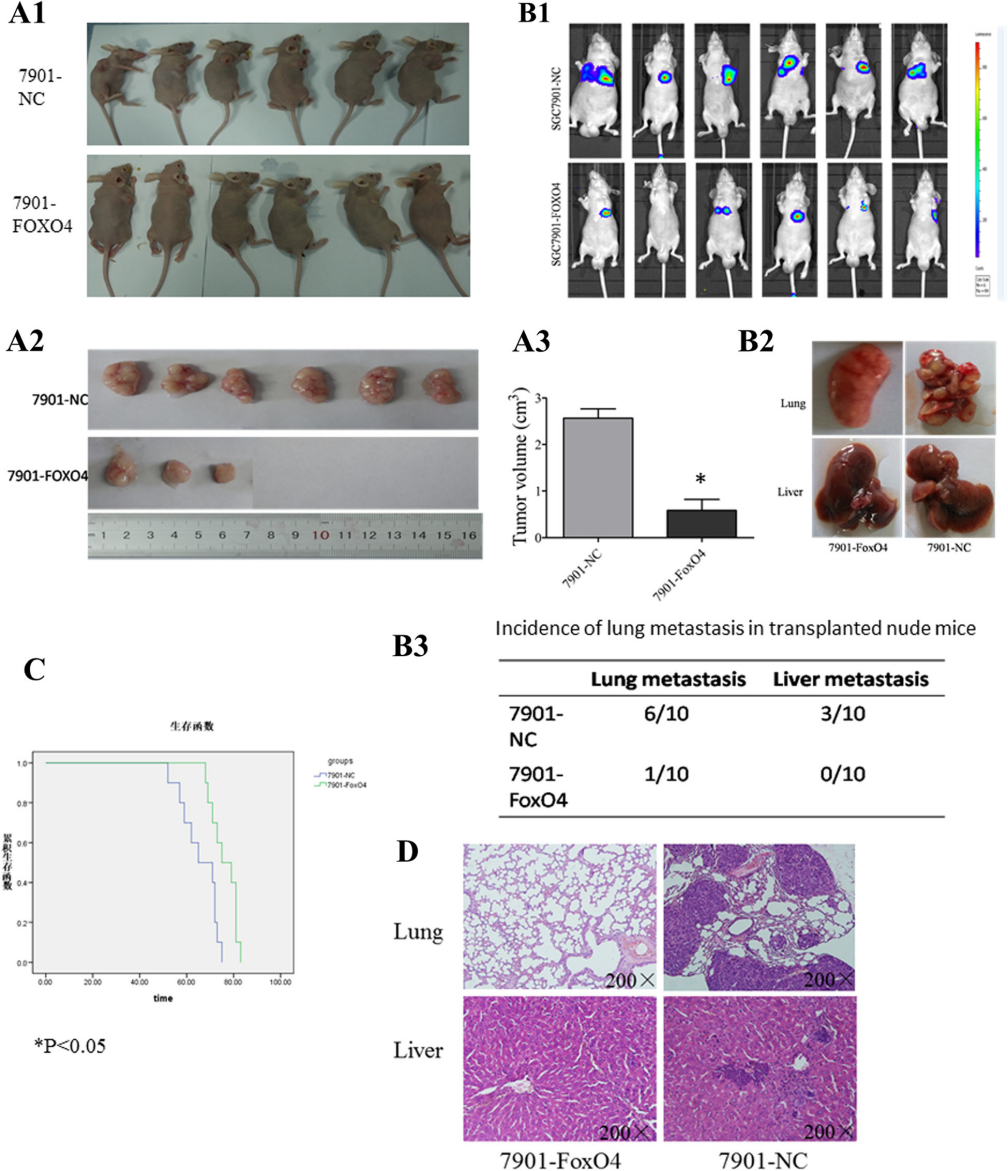
**In vivo proliferation and metastasis assay. (A1-A3)** SGC-7901 cells transfected with LV-FOXO4 or LV-control were transplanted under the skin. Six weeks later, tumors were more clearly seen in mice implanted with 7901-NC cells as compared to the 7901-FOXO4 groups: (6/6 in the 7901-NC groups and 3/6 in 7901-FOXO4 groups). The tumors were dissected and measured. **(B1-B3)** SGC-7901 cells transfected with LV-FOXO4 or LV-control were injected into the tail veins of nude mice. Ten weeks later, mice implanted with 7901-NC cells showed lung and liver metastases, whereas few metastases were detected in mice implanted with 7901-FOXO4 cells: (for lung metastasis, 6/10 in the 7901-NC groups and 1/10 in the 7901-FoxO4 groups, For liver metastasis, 3/10 in 7901-NC groups and 0/10 in 7901-FoxO4 groups. **(C)** Images showing representative hematoxylin and eosin staining of lung and liver tissue samples from the different experimental groups *P < 0.05. **(D)** Overall survival of the nude mice in each group.

To further confirm the effects of FOXO4 on the tumorigenesis of GC, a tumor formation assay was performed in nude mice. SGC7901-NC and SGC7901-FOXO4 cells were subcutaneously inoculated into the right upper back region of nude mice at a single site. Four weeks later, mice that were subcutaneously inoculated were sacrificed, the transplanted tumors were excised, and the tumor sizes were evaluated (Figure 4A1-A3, P < 0.05). The results revealed a significant decrease in the sizes of xenografts resulting from FOXO4 up-regulated cells.To further explore the role of FOXO4 in tumor metastasis in vivo, we implanted SGC7901-NC and SGC7901-FOXO4 cells into nude mice through the lateral tail vein. Representative bioluminescent imaging (BLI) of the different groups is shown in Figure 4B1. Histological analysis further confirmed that the incidence of lung and liver metastasis in the SGC7901-FOXO4 group was significantly decreased, compared to the SGC7901-NC group (Figure 4B2,B3). The number of lung metastatic nodules in the SGC7901-FOXO4 group was also reduced, compared to the SGC7901-NC group(data not shown). Liver and lung metastasis were further evidenced by hematoxylin and eosin staining (Figure 4C). Furthermore, the SGC7901-FOXO4 group nude mice demonstrated longer overall survival time compared to the SGC7901-NC group (Figure 4D). These data indicated that FOXO4 suppressed GC cell tumorigenesis and metastasis in vivo.

### Molecular mechanisms of FOXO4 in the metastasis of GC

**Figure 5:**
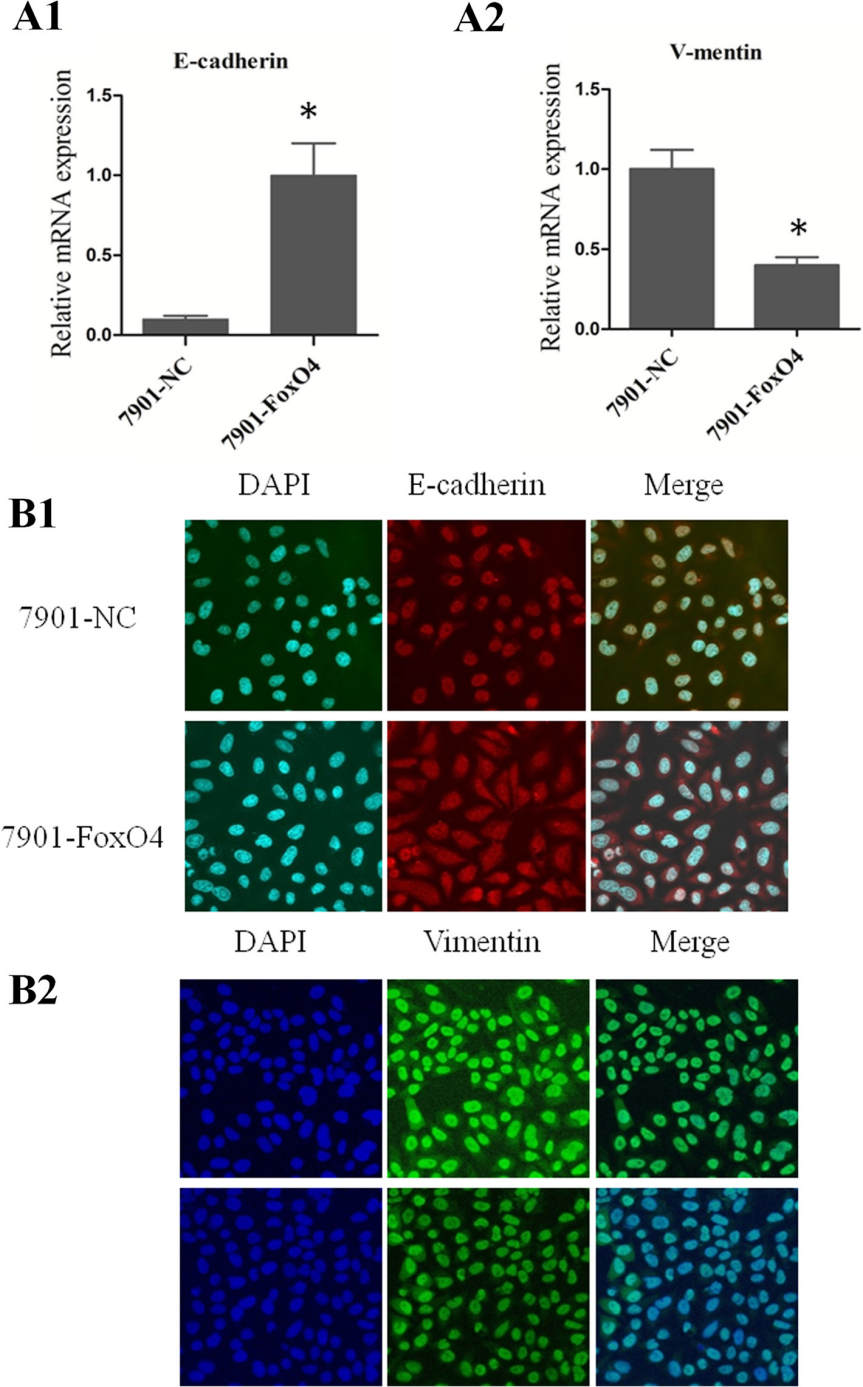
**FOXO4 inhibits EMT in GC cells. (A1-A2)** Real-time PCR showed up-regulated expression of epithelial markers (E-cadherin) and down-regulated expression of mesenchymal markers (vimentin) in 7901-FOXO4 cells. **(B1-B2)** Immunofluorescence staining showed up-regulated expression of epithelial markers (E-cadherin) and down-regulated expression of mesenchymal markers (vimentin) in 7901-FOXO4 cells.

To explore potential mechanisms for the role of FOXO4 in GC metastasis, we examined the expression of metastasis-related molecules, including E-cadherin, vimentin in SGC-7901-FOXO4 and SGC-7901-NC control cells using RT-PCR (Figure 5A1-A2). The results showed that FOXO4 overexpression markedly repressed the expression of vimentin, although no obvious alteration was observed for E-cadherin. The immunofluorescence confocal results also yielded similar conclusions (Figure 5B1-B2).

These data indicate that FOXO4 may partially influence GC cell metastasis by regulating EMT process, and additional molecular mechanisms will be studied in future work.

## Discussion

The forkhead box class O (FOXO) family of transcription factors is evolutionarily conserved and characterized by the so-called forkhead box DNA-binding domain. In mammals, the FOXO gene family consists of four members: FOXO1, FOXO3A, FOXO4, and FOXO6. Numerous studies have shown that FOXO proteins play an important role in a wide range of normal biological processes, including cellular proliferation, cell cycle arrest, stress response, and apoptosis [10,13,14], as well as in diseases such as cancer and diabetes mellitus [15]. However, there is little study reported about the role of FOXO4 plays in GC.

In the present study, we found the FOXO4 expression in non-tumorous tissues was consistently stronger than that of the GC samples, and GCs showed a lower expression level of FOXO4 in metastatic lesions compared to the corresponding primary tumor samples. The FOXO4 mRNA and protein expression levels were both reduced in various types of GC cell lines compared to the normal gastric mucosal epithelial cell line, suggesting that FOXO4 might serve as a negative regulator for GC. Additionally, elevated expression of FOXO4 expression inhibited tumor cell growth, invasion, and metastasis in vitro and in vivo, indicating that FOXO4 may play a role in GC progression and metastasis.

The mechanisms responsible for the impact of FOXO4 alterations on GC development and progression remain unclear. Several recent studies have indicated that FOXO regulates many aspects of cancer biology. For example, FOXO is normally restrained by the PI3K/Akt signaling pathway, which prevents FOXO translocation into the nucleus, and FOXO regulate transcriptional responses independently of direct DNA binding via association with a variety of unrelated transcription factors [16]. Our findings showed that FOXO4 induced significant G1 arrest and S phase reduction in GC cells, which indicated that FOXO4 inhibited GC proliferation may at least partly by the result of G1 cell-cycle arrest.

One critical step in the metastatic cascade is the process of epithelial to mesenchymal transition (EMT) [17,18]. During the EMT process, the expression of E-cadherin was often down-regulated, while which of vimentin often shows up-regulated [19]. FOXO4 may regulate EMT in gastric cancer. To test this hypothesis, we assessed the expressions of E-cadherin and vimentin in the cell models above. Although no obvious alteration was observed for E-cadherin, a dramatic decrease of vimentin expression was displayed in FOXO4 overexpression cells compared to the control cells, as indicated by immunofluorescent assay and qRT-PCR. These studies strongly suggest that FOXO4 might inhibit gastric cancer metastasis by regulating EMT.

## Conclusion

In conclusion, our study demonstrates a critical function of FOXO4 in the inhibition of GC proliferation and metastasis via the regulation of G1 cell-cycle arrest and EMT, suggests it may serve as a potential therapeutic target for gastric cancer.

## Abbreviations

FOXO4: Forkhead box O4; BSA: Bovine serum albumin; DAB: Diaminobenzidine; qRT-PCR: Real-time quantitative PCR; MTT: 3-[4,5-dimethylthiazol-2-yl]-2,5-diphenyl-tetrazolium bromide; PBS: Phosphate buffered saline; DMSO: Dimethyl sulfoxide.

## Competing interests

The authors declare that they have no competing interests.

## Authors’ contributions

YQS and DMF participated in the design of the study. LNS and XSL obtained all biopsies and carried out the immunohistochemical studies with the help of YZN. LNS and XQL carried out the immunohistochemical staining assessment. LNS, XQL and LFL performed the histological and functional examination, with the help from NC, RW and LFL. XQL and LNS performed the animal experiments and carried out the data analysis. LNS and XQL drafted the main manuscript, with contributions from the other authors. All authors read and approved the final manuscript.

## Pre-publication history

The pre-publication history for this paper can be accessed here:

http://www.biomedcentral.com/1471-2407/14/378/prepub

## Supplementary Material

Additional file 1: Table S1 Information of tissue array (human gastric adenocarcinoma with matched adjacent tissues). Click here for file

Additional file 2: Table S2 Clinical information of gastric cancer(GC) and corresponding lymph node metastasis specimens. Click here for file
