# Supplementary material for: The transcription factor FOXO4 is down-regulated and inhibits tumor proliferation and metastasis in gastric cancer
Source: BMC Cancer. 2014 May 28;14:378. doi: 10.1186/1471-2407-14-378 (PMC4063225; doi:10.1186/1471-2407-14-378)
Supplement: Additional file 1: Table S1 — Information of tissue array (human gastric adenocarcinoma with matched adjacent tissues). [file 1471-2407-14-378-S1.doc]

**Information of tissue array (human gastric adenocarcinoma with matched adjacent tissues)**

**Catalog No.:** C0124

**Product Name:** Human gastric adenocarcinoma with matched adjacent (75 cases) tissues array

**Species:** Human Rat Mouse Rabbit

**Tissue Type:** Adult Tumor Normal Cancerometastasis Adjacent

**Design Description:**

- Each core comes from 1 paraffin tissue block
- Gastric adenocarcinoma / Tubular adenocarcinoma / Mucinous adenocarcinoma
- Row : Column=10 : 15

**Total Cases:** 75

**Total Tissue Organs:** 1

**Total Tissue Blocks:** 150

**Diameter:** 1.0mm

**Thickness:** 3-5 μm

**Array:**


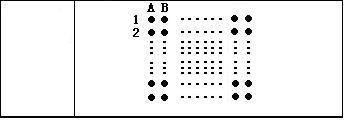


**Patient ID:**

|  | A | B | C | D | E | F | G | H | I | J | K | L | M | N | O |
| --- | --- | --- | --- | --- | --- | --- | --- | --- | --- | --- | --- | --- | --- | --- | --- |
|  | 551 | 553 | 554 | 556 | 557 | 560 | 562 | 564 | 566 | 567 | 569 | 571 | 578 | 579 | 581 |
|  | 551 | 553 | 554 | 556 | 557 | 560 | 562 | 564 | 566 | 567 | 569 | 571 | 578 | 579 | 581 |
|  | 583 | 584 | 688 | 586 | 587 | 588 | 590 | 591 | 592 | 593 | 595 | 596 | 597 | 598 | 599 |
|  | 583 | 584 | 688 | 586 | 587 | 588 | 590 | 591 | 592 | 593 | 595 | 596 | 597 | 598 | 599 |
|  | 601 | 608 | 609 | 612 | 689 | 618 | 620 | 621 | 622 | 623 | 625 | 626 | 627 | 629 | 631 |
|  | 601 | 608 | 609 | 612 | 689 | 618 | 620 | 621 | 622 | 623 | 625 | 626 | 627 | 629 | 631 |
|  | 637 | 638 | 640 | 641 | 643 | 647 | 649 | 650 | 651 | 652 | 655 | 656 | 659 | 660 | 666 |
|  | 637 | 638 | 640 | 641 | 643 | 647 | 649 | 650 | 651 | 652 | 655 | 656 | 659 | 660 | 661 |
|  | 664 | 665 | 666 | 690 | 669 | 670 | 672 | 673 | 674 | 675 | 677 | 679 | 681 | 685 | 687 |
|  | 664 | 665 | 666 | 690 | 669 | 670 | 672 | 670 | 674 | 675 | 677 | 679 | 681 | 685 | 687 |

**Array Design:**

|  | A | B | C | D | E | F | G | H | I | J | K | L | M | N | O |
| --- | --- | --- | --- | --- | --- | --- | --- | --- | --- | --- | --- | --- | --- | --- | --- |
|  | Stomach Cancer | Stomach Cancer | Stomach Cancer | Stomach Cancer | Stomach Cancer | Stomach Cancer | Stomach Cancer | Stomach Cancer | Stomach Cancer | Stomach Cancer | Stomach Cancer | Stomach Cancer | Stomach Cancer | Stomach Cancer | Stomach Cancer |
|  | Stomach Adjacent | Stomach Adjacent | Stomach Adjacent | Stomach Adjacent | Stomach Adjacent | Stomach Adjacent | Stomach Adjacent | Stomach Adjacent | Stomach Adjacent | Stomach Adjacent | Stomach Adjacent | Stomach Adjacent | Stomach Adjacent | Stomach Adjacent | Stomach Adjacent |
|  | Stomach Cancer | Stomach Cancer | Stomach Cancer | Stomach Cancer | Stomach Cancer | Stomach Cancer | Stomach Cancer | Stomach Cancer | Stomach Cancer | Stomach Cancer | Stomach Cancer | Stomach Cancer | Stomach Cancer | Stomach Cancer | Stomach Cancer |
|  | Stomach Adjacent | Stomach Adjacent | Stomach Adjacent | Stomach Adjacent | Stomach Adjacent | Stomach Adjacent | Stomach Adjacent | Stomach Adjacent | Stomach Adjacent | Stomach Adjacent | Stomach Adjacent | Stomach Adjacent | Stomach Adjacent | Stomach Adjacent | Stomach Adjacent |
|  | Stomach Cancer | Stomach Cancer | Stomach Cancer | Stomach Cancer | Stomach Cancer | Stomach Cancer | Stomach Cancer | Stomach Cancer | Stomach Cancer | Stomach Cancer | Stomach Cancer | Stomach Cancer | Stomach Cancer | Stomach Cancer | Stomach Cancer |
|  | Stomach Adjacent | Stomach Adjacent | Stomach Adjacent | Stomach Adjacent | Stomach Adjacent | Stomach Adjacent | Stomach Adjacent | Stomach Adjacent | Stomach Adjacent | Stomach Adjacent | Stomach Adjacent | Stomach Adjacent | Stomach Adjacent | Stomach Adjacent | Stomach Adjacent |
|  | Stomach Cancer | Stomach Cancer | Stomach Cancer | Stomach Cancer | Stomach Cancer | Stomach Cancer | Stomach Cancer | Stomach Cancer | Stomach Cancer | Stomach Cancer | Stomach Cancer | Stomach Cancer | Stomach Cancer | Stomach Cancer | Stomach Cancer |
|  | Stomach Adjacent | Stomach Adjacent | Stomach Adjacent | Stomach Adjacent | Stomach Adjacent | Stomach Adjacent | Stomach Adjacent | Stomach Adjacent | Stomach Adjacent | Stomach Adjacent | Stomach Adjacent | Stomach Adjacent | Stomach Adjacent | Stomach Adjacent | Stomach Adjacent |
|  | Stomach Cancer | Stomach Cancer | Stomach Cancer | Stomach Cancer | Stomach Cancer | Stomach Cancer | Stomach Cancer | Stomach Cancer | Stomach Cancer | Stomach Cancer | Stomach Cancer | Stomach Cancer | Stomach Cancer | Stomach Cancer | Stomach Cancer |
|  | Stomach Adjacent | Stomach Adjacent | Stomach Adjacent | Stomach Adjacent | Stomach Adjacent | Stomach Adjacent | Stomach Adjacent | Stomach Adjacent | Stomach Adjacent | Stomach Adjacent | Stomach Adjacent | Stomach Adjacent | Stomach Adjacent | Stomach Adjacent | Stomach Adjacent |

**Clinical Information of Human gastric adenocarcinoma with matched adjacent (75 cases) tissues array**

| **No.** | **Position** | **Specimen Description & Location** | **Pathological Diagnosis** | **Grade** | **TNM** | **Status** | **Overall Survival (Months)** |
| --- | --- | --- | --- | --- | --- | --- | --- |
|  | <1A> | Gastric body | Adenocarcinoma | Ⅱ | T3N1M0 | Died | 23 |
|  | 1B | Lesser curvature of stomach | Adenocarcinoma | Ⅱ | T2N0M0 | Survival | 51 |
|  | <1C> | Fundus of stomach | Adenocarcinoma | Ⅲ | T2N0M0 | Died | 9 |
|  | 1D | Stomach | Tubular adenocarcinoma |  | T3N1M0 | Died | 26 |
|  | 1E | Antrum of stomach | Adenocarcinoma | Ⅱ-Ⅲ | T3N1M0 | Survival | 51 |
|  | <1F> | Gastric body | Adenocarcinoma | Ⅰ-Ⅱ | T2N0M0 | Died | 39 |
|  | <1G> | Gastric body | Adenocarcinoma | Ⅱ-Ⅲ | T3N1M0 | Died | 10 |
|  | 1H | Antrum of stomach | Mucinous adenocarcinoma |  | T2N0M0 | Died | 9 |
|  | 1I | Lesser curvature of stomach | Adenocarcinoma | Ⅲ | T3N1M0 | Died | 6 |
|  | 1J | Fundus of stomach | Adenocarcinoma | Ⅲ | T3N1M0 | Died | 43 |
|  | 1K | Lesser curvature of stomach | Adenocarcinoma | Ⅲ | T2N0M0 | Died | 39 |
|  | 1L | Fundus of stomach | Adenocarcinoma | Ⅱ-Ⅲ | T2N0M0 | Died | 16 |
|  | 1M | Lesser curvature of antrum | Ulcer type adenocarcinoma | Ⅲ | T3N1M0 | Died | 35 |
|  | 1N | Gastric body | Mucinous adenocarcinoma | Ⅲ | T2N0M0 | Died | 5 |
|  | 1O | Antrum of stomach | Adenocarcinoma | Ⅲ | T3N1M0 | Died | 14 |
|  | 2A | Adjacent of 1A | Gastric mucosa |  |  | Died | 23 |
|  | 2B | Adjacent of 1B | Chronic inflammation |  |  | Survival | 51 |
|  | 2C | Adjacent of 1C | Chronic inflammation |  |  | Died | 9 |
|  | 2D | Adjacent of 1D | Gastric mucosa |  |  | Died | 26 |
|  | 2E | Adjacent of 1E | Gastric mucosa |  |  | Survival | 51 |
|  | 2F | Adjacent of 1F | Gastric mucosa |  |  | Died | 39 |
|  | 2G | Adjacent of 1G | Chronic inflammation |  |  | Died | 10 |
|  | 2H | Adjacent of 1H | Gastric mucosa |  |  | Died | 9 |
|  | 2I | Adjacent of 1I | Gastric mucosa |  |  | Died | 6 |
|  | 2J | Adjacent of 1J | Gastric mucosa |  |  | Died | 43 |
|  | 2K | Adjacent of 1K | Gastric mucosa |  |  | Died | 39 |
|  | 2L | Adjacent of 1L | Gastric mucosa |  |  | Died | 16 |
|  | 2M | Adjacent of 1M | Chronic inflammation |  |  | Died | 35 |
|  | 2N | Adjacent of 1N | Gastric mucosa |  |  | Died | 5 |
|  | 2O | Adjacent of 1O | Gastric mucosa |  |  | Died | 14 |
|  | 3A | Greater curvature of gastric body | Adenocarcinoma | Ⅲ | T2N0M0 | Died | 11 |
|  | 3B | Antrum of stomach | Adenocarcinoma | Ⅰ | T3N1M0 | Survival | 50 |
|  | 3C | Gastric body | Adenocarcinoma |  | T3N1M0 | Died | 28 |
|  | 3D | Fundus of stomach | Adenocarcinoma | Ⅱ-Ⅲ | T3N1M0 | Died | 32 |
|  | 3E | Lesser curvature of stomach | Adenocarcinoma | Ⅲ | T2N0M0 | Survival | 50 |
|  | 3F | Cardia of stomach | Adenocarcinoma | Ⅱ | T3N1M0 | Survival | 50 |
|  | 3G | Cardia of stomach | Adenocarcinoma | Ⅱ-Ⅲ | T2N0M0 | Died | 24 |
|  | 3H | Antrum of stomach | Mucinous adenocarcinoma |  | T3N1M0 | Survival | 49 |
|  | 3I | Pylorus of stomach | Adenocarcinoma | Ⅱ | T3N1M0 | Died | 21 |
|  | 3J | Lesser curvature of the distal stomach | Adenocarcinoma | Ⅲ | T3N1M0 | Survival | 49 |
|  | 3K | Gastric body | Adenocarcinoma | Ⅲ | T3N1M0 | Died | 44 |
|  | 3L | Cardia of stomach | Adenocarcinoma | Ⅱ | T3N1M0 | Died | 27 |
|  | 3M | Fundus of stomach | Adenocarcinoma | Ⅱ-Ⅲ | T2N0M0 | Died | 10 |
|  | 3N | Greater curvature side of stomach | Adenocarcinoma | Ⅲ | T3N1M0 | Survival | 49 |
|  | 3O | Lesser curvature of stomach | Adenocarcinoma | Ⅲ | T3N1M0 | Survival | 49 |
|  | 4A | Adjacent of 3A | Gastric mucosa |  |  | Died | 11 |
|  | 4B | Adjacent of 3B | Chronic inflammation |  |  | Survival | 50 |
|  | 4C | Adjacent of 3C | Chronic inflammation |  |  | Died | 28 |
|  | 4D | Adjacent of 3D | Gastric mucosa |  |  | Died | 32 |
|  | 4E | Adjacent of 3E | Gastric mucosa |  |  | Survival | 50 |
|  | 4F | Adjacent of 3F | Gastric mucosa |  |  | Survival | 50 |
|  | 4G | Adjacent of 3G | Gastric mucosa, partly infiltrated by cancer cells |  |  | Died | 24 |
|  | 4H | Adjacent of 3H | Chronic inflammation |  |  | Survival | 49 |
|  | 4I | Adjacent of 3I | Gastric mucosa, partly infiltrated by inflammatory cells |  |  | Died | 21 |
|  | 4J | Adjacent of 3J | Gastric mucosa |  |  | Survival | 49 |
|  | 4K | Adjacent of 3K | Gastric mucosa |  |  | Died | 44 |
|  | 4L | Adjacent of 3L | Gastric mucosa |  |  | Died | 27 |
|  | 4M | Adjacent of 3M | Gastric mucosa, partly infiltrated by inflammatory cells |  |  | Died | 10 |
|  | 4N | Adjacent of 3N | Gastric mucosa |  |  | Survival | 49 |
|  | 4O | Adjacent of 3O | Gastric mucosa |  |  | Survival | 49 |
|  | 5A | Gastric body | Mucinous adenocarcinoma |  | T3N1M0 | Died | 43 |
|  | 5B | Lesser curvature of antrum | Adenocarcinoma | Ⅲ | T2N0M0 | Survival | 49 |
|  | 5C | Cardia of stomach | Adenocarcinoma | Ⅱ-Ⅲ | T2N0M0 | Died | 35 |
|  | 5D | Stomach | Adenocarcinoma | Ⅲ | T3N1M0 | Survival | 49 |
|  | 5E | Antrum of stomach | Tubular adenocarcinoma | Ⅱ-Ⅲ | T2N0M0 | Survival | 49 |
|  | 5F | Fundus of stomach | Adenocarcinoma | Ⅱ-Ⅲ | T3N1M0 | Died | 2 |
|  | 5G | Lesser curvature of gastric cardia | Adenocarcinoma | Ⅱ-Ⅲ | T3N1M0 | Died | 24 |
|  | 5H | Antrum of stomach | Mushroom type adenocarcinoma | Ⅱ | T3N1M0 | Died | 46 |
|  | 5I | Lesser curvature of stomach | Adenocarcinoma | Ⅲ | T2N0M0 | Died | 15 |
|  | 5J | Lesser curvature of stomach | Adenocarcinoma | Ⅱ | T3N1M0 | Survival | 49 |
|  | 5K | Antrum of stomach | Adenocarcinoma | Ⅱ | T3N1M0 | Died | 34 |
|  | 5L | Lesser curvature of stomach | Adenocarcinoma | Ⅱ | T3N1M0 | Survival | 48 |
|  | 5M | Gastric body | Adenocarcinoma | Ⅲ | T3N1M0 | Died | 42 |
|  | 5N | Pylorus of stomach | Ulcer type mucinous adenocarcinoma |  | T3N1M0 | Survival | 48 |
|  | 5O | Fundus of stomach | Adenocarcinoma | Ⅲ | T3N1M0 | Survival | 48 |
|  | 6A | Adjacent of 5A | Gastric mucosa |  |  | Died | 43 |
|  | 6B | Adjacent of 5B | Gastric mucosa |  |  | Survival | 49 |
|  | 6C | Adjacent of 5C | Chronic inflammation |  |  | Died | 35 |
|  | 6D | Adjacent of 5D | Gastric mucosa, partly infiltrated by inflammatory cells |  |  | Survival | 49 |
|  | 6E | Adjacent of 5E | Gastric mucosa |  |  | Survival | 49 |
|  | 6F | Adjacent of 5F | Gastric mucosa |  |  | Died | 2 |
|  | 6G | Adjacent of 5G | Chronic inflammation |  |  | Died | 24 |
|  | 6H | Adjacent of 5H | Gastric mucosa |  |  | Died | 46 |
|  | 6I | Adjacent of 5I | Gastric mucosa |  |  | Died | 15 |
|  | 6J | Adjacent of 5J | Gastric mucosa |  |  | Survival | 49 |
|  | 6K | Adjacent of 5K | Chronic inflammation |  |  | Died | 34 |
|  | 6L | Adjacent of 5L | Gastric mucosa |  |  | Survival | 48 |
|  | 6M | Adjacent of 5M | Gastric mucosa |  |  | Died | 42 |
|  | 6N | Adjacent of 5N | Gastric mucosa |  |  | Survival | 48 |
|  | 6O | Adjacent of 5O | Chronic inflammation |  |  | Survival | 48 |
|  | 7A | Lesser curvature of stomach | Adenocarcinoma | Ⅱ-Ⅲ | T3N1M0 | Survival | 48 |
|  | 7B | Antrum and body of stomach | Adenocarcinoma | Ⅲ | T2N0M0 | Died | 25 |
|  | 7C | Antrum of stomach | Adenocarcinoma | Ⅲ | T3N1M0 | Died | 5 |
|  | 7D | Gastric body | Adenocarcinoma | Ⅱ | T3N1M0 | Survival | 48 |
|  | 7E | Lesser curvature of stomach | Adenocarcinoma | Ⅲ | T3N1M0 | Survival | 48 |
|  | 7F | Gastric body | Adenocarcinoma | Ⅲ | T2N0M0 | Survival | 48 |
|  | 7G | Lesser curvature of stomach | Adenocarcinoma | Ⅱ-Ⅲ | T2N0M0 | Died | 43 |
|  | 7H | Gastric body | Adenocarcinoma | Ⅲ | T3N1M0 | Died | 15 |
|  | 7I | Antrum of stomach | Ulcer type adenocarcinoma | Ⅲ | T3N1M0 | Died | 15 |
|  | 7J | Fundus of stomach | Adenocarcinoma | Ⅲ | T3N1M0 | Survival | 47 |
|  | 7K | Pylorus of stomach | Adenocarcinoma | Ⅱ | T2N0M0 | Died | 36 |
|  | 7L | Antrum of stomach | Adenocarcinoma | Ⅱ | T3N1M0 | Survival | 47 |
|  | 7M | Antrum of stomach | Adenocarcinoma | Ⅱ-Ⅲ | T2N0M0 | Died | 11 |
|  | 7N | Gastric body | Adenocarcinoma | Ⅱ | T3N1M0 | Survival | 47 |
|  | 7O | Lesser curvature of stomach | Adenocarcinoma | Ⅱ-Ⅲ | T3N1M0 | Survival | 47 |
|  | 8A | Adjacent of 7A | Chronic inflammation |  |  | Survival | 48 |
|  | 8B | Adjacent of 7B | Gastric mucosa |  |  | Died | 25 |
|  | 8C | Adjacent of 7C | Chronic inflammation |  |  | Died | 5 |
|  | 8D | Adjacent of 7D | Gastric mucosa |  |  | Survival | 48 |
|  | 8E | Adjacent of 7E | Gastric mucosa |  |  | Survival | 48 |
|  | 8F | Adjacent of 7F | Gastric mucosa |  |  | Survival | 48 |
|  | 8G | Adjacent of 7G | Gastric mucosa |  |  | Died | 43 |
|  | 8H | Adjacent of 7H | Chronic inflammation |  |  | Died | 15 |
|  | 8I | Adjacent of 7I | Gastric mucosa |  |  | Died | 15 |
|  | 8J | Adjacent of 7J | Gastric mucosa |  |  | Survival | 47 |
|  | 8K | Adjacent of 7K | Gastric mucosa |  |  | Died | 36 |
|  | 8L | Adjacent of 7L | Gastric mucosa |  |  | Survival | 47 |
|  | 8M | Adjacent of 7M | Gastric mucosa |  |  | Died | 11 |
|  | 8N | Adjacent of 7N | Gastric mucosa |  |  | Survival | 47 |
|  | 8O | Adjacent of 7O | Gastric mucosa |  |  | Survival | 47 |
|  | 9A | Gastric body | Adenocarcinoma | Ⅱ | T3N1M0 | Survival | 47 |
|  | 9B | Gastric body | Adenocarcinoma | Ⅱ | T3N1M0 | Survival | 47 |
|  | 9C | Fundus of stomach | Adenocarcinoma | Ⅱ-Ⅲ | T2N0M0 | Died | 22 |
|  | 9D | Lesser curvature of stomach | Adenocarcinoma | Ⅲ | T3N1M0 | Died | 2 |
|  | 9E | Cardia of stomach | Adenocarcinoma | Ⅱ | T3N1M0 | Survival | 47 |
|  | 9F | Fundus of stomach | Adenocarcinoma | Ⅲ | T3N1M0 | Died | 24 |
|  | 9G | Antrum of stomach | Adenocarcinoma | Ⅰ-Ⅱ | T2N0M0 | Survival | 43 |
|  | 9H | Pylorus of stomach | Adenocarcinoma | Ⅰ | T3N1M0 | Died | 17 |
|  | 9I | Fundus of stomach | Adenocarcinoma | Ⅰ | T3N1M0 | Died | 27 |
|  | 9J | Fundus of stomach | Adenocarcinoma | Ⅱ | T3N1M0 | Died | 31 |
|  | 9K | Lesser curvature of stomach | Adenocarcinoma | Ⅰ | T2N0M0 | Survival | 43 |
|  | 9L | Fundus of stomach | Adenocarcinoma | Ⅱ-Ⅲ | T3N1M0 | Died | 35 |
|  | 9M | Gastric body | Mucinous adenocarcinoma |  | T3N1M0 | Died | 13 |
|  | 9N | Antrum of stomach | Ulcer type adenocarcinoma | Ⅰ | T3N1M0 | Died | 11 |
|  | 9O | Fundus of stomach | Adenocarcinoma | Ⅲ | T3N1M0 | Died | 39 |
|  | 10A | Adjacent of 9A | Gastric mucosa |  |  | Survival | 47 |
|  | 10B | Adjacent of 9B | Chronic inflammation |  |  | Survival | 47 |
|  | 10C | Adjacent of 9C | Gastric mucosa |  |  | Died | 22 |
|  | 10D | Adjacent of 9D | Gastric mucosa |  |  | Died | 2 |
|  | 10E | Adjacent of 9E | Gastric mucosa |  |  | Survival | 47 |
|  | 10F | Adjacent of 9F | Chronic inflammation |  |  | Died | 24 |
|  | 10G | Adjacent of 9G | Chronic inflammation |  |  | Survival | 43 |
|  | 10H | Adjacent of 9H | Gastric mucosa |  |  | Died | 17 |
|  | 10I | Adjacent of 9I | Chronic inflammation |  |  | Died | 27 |
|  | 10J | Adjacent of 9J | Gastric mucosa |  |  | Died | 31 |
|  | 10K | Adjacent of 9K | Chronic inflammation |  |  | Survival | 43 |
|  | 10L | Adjacent of 9L | Gastric mucosa |  |  | Died | 35 |
|  | 10M | Adjacent of 9M | Gastric mucosa |  |  | Died | 13 |
|  | 10N | Adjacent of 9N | Chronic inflammation |  |  | Died | 11 |
|  | 10O | Adjacent of 9O | Chronic inflammation |  |  | Died | 39 |
